# Supplementary material for: Validity and reliability of velocity and power measures provided by the Vitruve linear position transducer
Source: PLoS One. 2024 Oct 24;19(10):e0312348. doi: 10.1371/journal.pone.0312348 (PMC11500865; doi:10.1371/journal.pone.0312348)
Supplement: Attachment — Submitted filename: Response to reviewers.docx [file pone.0312348.s003.docx]

**Reviewer #1:**

**COMMENT:**

The Vitruve clearly didn't measure what it said it would measure. I understand you likely don't want to make too strong of statements in the paper, but the data wasn't valid for MV, PP, MP, and PV. We also can see that it more or less only worked ok with a Smith machine, which few people use in real life. Considering all of this, I wouldn't focus on the fact that the device cannot be used interchangeably with other devices, but I would simply advise against using it. It doesn't work good enough for academic researchers to recommend its use "even if you only use it and no other equipment". If readers don't know that you can't use data from different devices by now, they wouldn't learn anything new by stating that in this paper. Thus, please rework the discussion and conclusions to indicate that the device should not be used (not even "if you accept X amount of error"). In summary, I would like to see stronger statements. If you bought this device and used it, would you be entirely happy with the data? If so, no need to have stronger statements. However, if you wouldn't be pleased with the device, other people wouldn't either. Therefore, the story goes beyond the data.

**RESPONSE:**

The authors would like to thank the reviewer for their valuable feedback. We agree with the reviewer that our conclusions may not have fully expressed our findings. Your observations align with those made by the second reviewer, indicating that this aspect needed improvement. We have addressed this issue throughout the manuscript. Specifically, please review the revised conclusion section:

“Considering GymAware as a reference point, Vitruve was not valid for measuring velocity and power outcomes. Acceptable validity was observed only for PV in the Smith machine back squat, while the rest of the variables were mostly inaccurate across relative loads and regardless of the exercise mode. On the other hand, Vitruve users should note that MV and PV displayed superior reliability compared to MP and PP. Additionally, all variables measured by Vitruve were more reliable in the Smith machine compared to the free-weight back squat exercise mode. Finally, practitioners are encouraged to consider the SDD reported here for MV, PV, MP, and PP when interpreting meaningful changes in performance”

**Reviewer #2:**

**COMMENT:**
The study determined the validity and between-day reliability of the mean velocity, peak velocity, mean power, and peak power provided by the Vitruve device at different relative loads in the free-weight and Smith machine back squat. In general, the results showed that the Vitruve did not meet the validity criteria for almost all variables analyzed but presented acceptable reliability for all variables analyzed. I congratulate the authors for their time and effort in conducting this study. The experimental procedures seem rigorous, but the introduction, discussion, statistical analysis, and reporting of results can be improved. The reasons for conducting the study and its pertinence should also be reinforced (as I understood, at least two studies have already explored this theme). Furthermore, the small sample size of fourteen male sports science students should be adequately justified in the methods and addressed in a limitations section. Finally, although the Viturve seems reliable, it is not valid, especially when measuring the mean velocity. Therefore, I cannot entirely agree with the authors' statement that Vitruve seems useful for resistance training, monitoring, and prescription. This critical information should be well revised throughout the manuscript. Major revisions should be made. Below, I provide some comments and suggestions. Best regards.

**RESPONSE:**

The authors wish to express their gratitude for the constructive feedback provided. These insights have substantially enhanced the quality of the manuscript. Specifically, we have elucidated the novelty of the study in the introduction and revised the conclusions to address the concerns regarding the validity of the Vitruve device. The methodological concerns raised by the reviewer have been addressed and discussed in our response. Additionally, a limitations section has been included as requested. Thank you once again for your valuable feedback.

**COMMENT:**
ABSTRACT
LL57-58: Please rewrite the sentence for clarity.

**RESPONSE:**

Thanks for the suggestion. We have rewritten the sentence for clarity as follows: “Vitruve displayed both fixed and proportional bias for certain relative loads across all variables.”

**COMMENT:**
LL63-66: According to your data, Vitruve is reliable but does not present valid measures of velocity and power across a broad range of relative loads. In other words, Vitruve is consistent but does not accurately measure what it is meant to measure, especially the mean velocity (one of the most reported variables in velocity-based RT interventions). Therefore, if a device is not valid, it is useless. Please consider rewriting the sentence.
**RESPONSE:**

The sentence has been rewritten to reflect the results obtained more accurately:

Considering GymAware as a reference point, Vitruve was not valid for measuring velocity and power outcomes. Acceptable validity was observed only for PV in the Smith machine back squat, while the rest of the variables were mostly inaccurate across relative loads and regardless of the exercise mode.

**COMMENT:**
INTRODUCTION
- LL72-77: Please consider citing the review conducted by Badillo et al. (2022), as it addresses the core concepts of velocity-based resistance training (10.1186/s40798-022-00513-z).

**RESPONSE:**

Thanks for the suggested reference. We agree that this review outlines the core concepts of VBT and should be cited.

**COMMENT:**
- LL78: "Different velocity-monitoring systems are available nowadays." Please consider citing some studies.

**RESPONSE:**

Thanks for the suggestion. The following studies have been added which analyses the validity and reliability of the main commercially available devices:

Perez-Castilla, A., Piepoli, A., Delgado-García, G., Garrido-Blanca, G., & García-Ramos, A. (2019). Reliability and concurrent validity of seven commercially available devices for the assessment of movement velocity at different intensities during the bench press. The Journal of Strength & Conditioning Research, 33(5), 1258-1265.

Weakley, J., Morrison, M., García-Ramos, A., Johnston, R., James, L., & Cole M.H. (2021) The Validity and Reliability of Commercially Available Resistance Training Monitoring Devices: A Systematic Review. Sports Medicine 51:443–502.

**COMMENT:**
- LL79: "...valid and reliable devices providing barbell velocity data are needed". Please consider adding a brief definition of validity and reliability.

**RESPONSE:**

We have added brief definitions of validity and reliability to clarify these concepts for the readers:

- Validity: the accuracy of the device in measuring what it is supposed to measure.
- Reliability: the consistency of the measurements under consistent conditions.

**COMMENT:**
- LL84-86: I would complement this sentence by adding linear velocity transducers.

**RESPONSE:**

Thanks for the suggestion. The sentence has been modified accordingly:

For instance, sports scientists and strength and conditioning professionals frequently use linear position or velocity transducers. These devices utilize a cable attached to the barbell to determine the vertical displacement and calculate barbell velocity. This can be achieved through the inverse dynamics approach (linear position transducer), or by directly measuring velocity via electrical signals proportional to the cable's extension velocity (linear velocity transducer) (6).

**COMMENT:**
- LL88-90: Please consider citing the review conducted by Moreno-Villanueva on this topic (10.1080/14763141.2021.1988136).

**RESPONSE:**

Thanks for the reference, it has been added to the text.

**COMMENT:**
- LL96-107: These lines are important as they formulate the research problem. However, what is the main difference between the current study and those previously conducted? This information should be much more explicit so that readers can understand the current study's need and novelty.

**RESPONSE:**

Thanks for the suggestion to clarify this point. We believe that several methodological issues in current studies about Vitruve highlight the need for and novelty of our study.

First of all, only two studies have been conducted on Vitruve, which in itself establishes the need for our study. Secondly, the study by Külkamp et al. determined the validity of Vitruve for monitoring barbell displacement from pre-defined heights set on a Smith machine. This method lacks ecological validity as it does not consider free modes of exercise (i.e., free-weight back squat), where the horizontal motion of the lift could affect the device’s calculations. Our study aims to address this gap. Lastly, Killgallon et al. assessed the reliability of MPV, MV, and PV in the free-weight back squat. We deemed it necessary to investigate the reliability of the remaining measures (MP, PP) in various exercise modes, including the Smith machine. Additionally, Killgallon et al. determined the validity of Vitruve for estimating 1RM. Therefore, we aimed to determine its agreement with a well-established linear position transducer (GymAware) to provide a more comprehensive view of its validity.

These knowledge gaps are discussed in paragraphs 4 and 5. However, to reinforce these issues for readers, we have included a brief statement:

“Based on the aforementioned points, several knowledge gaps need to be addressed. These include determining the validity of Vitruve across various exercise modes (e.g., free-weight and Smith machine back squat), evaluating the validity of additional metrics (i.e., mean power [MP] and peak power [PP]), and assessing its level of agreement with other reference linear position transducers”.

**COMMENT:**
- LL108-110: Please add references.

**RESPONSE:**

The following study has been added:

Pérez-Castilla, A., Miras-Moreno, S., García-Vega, A. J., & García-Ramos, A. (2022). The placement of linear transducers affects the magnitude but not the intra-session reliability of kinematic variables during the bench press exercise. Isokinetics and Exercise Science, 30(4), 357-366.

**COMMENT:**
- LL115: Why the GymAware and not a different device? The reason(s) for introducing this device in the narrative must be well-defined.

**RESPONSE:**

Thanks for the suggestion to clarify this point. The validity of GymAware has been consolidated in different studies and readers can find this information on L123-129:

“However, the GymAware linear position transducer incorporates a sensor that measures the angle of the cable, which supposedly corrects the cable displacement according to the horizontal motion of the lift using basic trigonometry (11). Considering the proven validity of GymAware compared to gold-standard 3D motion capture systems (12–14), it would be of interest to examine the concurrent validity of Vitruve with respect to GymAware in different exercise modes (e.g., free-weight and Smith machine back squat).”

Please, also note that both the GymAware and T-Force are recommended as gold-standards in studies designed to validate other velocity monitoring devices. This fact is justified due to i) the comparable reliability between both devices and ii) the high degree of agreement between both devices, especially for PV (see Janicijevic et al., (2023) for further details).

Janicijevic, D., García-Ramos, A., Lamas-Cepero, J. L., García-Pinillos, F., Marcos-Blanco, A., Rojas, F. J., ... & Pérez-Castilla, A. (2023). Comparison of the two most commonly used gold-standard velocity monitoring devices (GymAware and T-Force) to assess lifting velocity during the free-weight barbell back squat exercise. *Proceedings of the Institution of Mechanical Engineers, Part P: Journal of Sports Engineering and Technology*, *237*(3), 205-212.

**COMMENT:**
- LL122-124: What is the need/relevance to include power variables? Are the estimates of power accurate in these devices? Previous authors have alerted for inaccurate power measures when using barbell kinematics (10.1519/JSC.0b013e31822e7b48). Considering that a force plate must be used for accurate power estimates, I question the utility of reporting power variables in the study.

**RESPONSE:**

Thank you for the suggestion to discuss this point. The reality is that athletes and strength and conditioning professionals use this metric in their sessions. Therefore, we deemed it necessary to address the reliability of the power it measures from the barbell and compare it with GymAware, as factors such as sampling rate and data manipulation by the software could cause discrepancies, which we aim to investigate.

**COMMENT:**
- LL130: Please consider formulating the study's hypotheses based on previous research.

**RESPONSE:**

Thanks for the suggestion. The following has been added to the text:

“Based on current knowledge about Vitruve device (8,16), it is hypothesized that the MV and MP may be underestimated. However, it is expected that all variables will display acceptable reliability.”

**COMMENT:**
MATERIALS AND METHODS

- LL152: "Fourteen male sports science students...". I recommend the authors present a reasonable sample size estimation based on an expected reliability value. Please also indicate the statistical power of the study.

**RESPONSE:**

Thanks for the suggestion. The sample was recruited based on convenience. Therefore, a post-hoc power analysis has been performed (which is not ideal, and in fact, is discouraged practice):

A post hoc power analysis was performed using G*Power (Version 3.1) with an effect size (ES) of 1.0 (the minimal difference observed for MV) and an alpha level (α) of 0.05. The analysis revealed a statistical power of 0.93.

**COMMENT:**
- LL162-187: Please add references for these procedures.

**RESPONSE:**

We inquired about our participants' warm-up routines and subsequently developed the proposed warm-up protocol. Note that a specific warm-up seems to enhance neuromuscular action (i.e., higher movement velocity) during the first training repetitions (Ribeiro et al., 2021). In this way, the subjects completed 10 back squat repetitions with a free-weight barbell (20 kg) and with the unloaded Smith machine barbell (19 kg) as part of specific warm-up even though the different relative loads were applied in an incremental order (i.e., 20, 40, 60, 80, 90% 1RM).

Ribeiro, B., Pereira, A., Alves, A. R., Neves, P. P., Marques, M. C., Marinho, D. A., & Neiva, H. P. (2021). Specific warm-up enhances movement velocity during bench press and squat resistance training. *Journal of Men’s Health*, *18*, 1-8.

**COMMENT:**
- LL188-195: Please add references for these procedures.

**RESPONSE:**

The following study has been added:

Jukic, I., García-Ramos, A., Malecek, J., Omcirk, D., & Tufano, J. J. (2022). Magnitude and reliability of velocity and power variables during deadlifts performed with and without lifting straps. Journal of Strength and Conditioning Research, 36(5), 1177-1184.

**COMMENT:**
- LL198-202: Was the absolute load adjusted so that the velocity achieved corresponded to the relative intensity? How can the authors guarantee that the velocities reached during sessions correspond to the actual velocities of the participants? This aspect should be clear, considering the typical fluctuations of 1RM from day to day, thus affecting all relative loads.

**RESPONSE:**

Thanks for the suggestion to clarify this point. We opted to fix the 1RM determined in the first week for the subsequent sessions as we considered it the most reliable method for determining the relative loads in the short term.

In this regard, Banyard et al. showed that the actual 1RM in the free-weight back squat was very stable between 3 trials (-5.6 to 4.8%) meanwhile the predicted 1RM through the load-velocity relationship (-5.6 to 27.8%) was more variable. Likewise, Alexander et al. showed that the free-weight back squat 1RM was less sensitive to the fatigue caused by a training session on the following days, but the squat velocity was altered.

Finally, this should not significantly affect the aspect of the study testing the validity of the devices, as both devices were used simultaneously, meaning that any potential error would impact both devices equally.

Banyard, H. G., Nosaka, K., & Haff, G. G. (2017). Reliability and validity of the load–velocity relationship to predict the 1RM back squat. The Journal of Strength & Conditioning Research, 31(7), 1897-1904.

Vernon, A., Joyce, C., & Banyard, H. G. (2020). Readiness to train: Return to baseline strength and velocity following strength or power training. International Journal of Sports Science & Coaching, 15(2), 204-211.

**COMMENT:**
- LL229-231: The CV (%) is more commonly associated with reliability, as it indicates the degree of variation or dispersion between measures (i.e., the consistency of a measurement device).

**RESPONSE:**

Thank you for your feedback. We agree that this topic is open to debate, and there is no consensus on which measures of validity should be used. In this case, we have based our selection on the statistical analysis performed in previous studies:

Weakley, J., Morrison, M., García-Ramos, A., Johnston, R., James, L., & Cole, M.H. (2021). The Validity and Reliability of Commercially Available Resistance Training Monitoring Devices: A Systematic Review. Sports Medicine, 51, 443–502.

Incorporating different metrics into this section could provide researchers with a valuable way to contrast their results.

**COMMENT:**
- In the statistical analysis subsection, I recommend that the authors calculate and report the intraclass correlation coefficient (ICC), Lin's concordance correlation coefficient (CCC), and linear regression (r2 and SEE) to increase the robustness of their analysis. These tests have been consistently used in similar research; thus, they should be reported for further comparisons.

**RESPONSE:**

Thank you for the suggestion to improve this section. Absolute reliability measures such as R² and SEE can be found in Figure 2. However, we decided to exclude relative reliability measures for the following reasons. Relative reliability, often represented by the Intraclass Correlation Coefficient (ICC), assesses how consistently individuals maintain their rank within a sample across repeated measurements. The ICC is typically greater for power measures since these are dependent on the absolute load lifted and the body mass of the individuals, unlike velocity measures, which tend to be similar (though not identical) between individuals at a given relative load (% 1RM). Therefore, while both types of reliability are commonly considered, absolute measures are more crucial for determining the reliability of each Vitruve metric, as illustrated in Figure 3.

**COMMENT:**
- LL242-245: Please briefly describe how the CV ratio was calculated.

**RESPONSE:**

Thanks for the suggestion. The reliability of the metrics, exercise modes, and devices was compared by averaging the CV across the loads and modes, and calculating the CV ratios, defined as (Highest CV)/(Lowest CV). To interpret the magnitude of differences between two CVs (e.g., Smith machine vs. free-weight back squat; mean velocity vs. mean power), a criterion for the smallest important ratio was established as higher than 1.15 (4). This has been described in the text.

**COMMENT:**
RESULTS
This section must be updated to integrate the results of the tests recommended before (ICC, CCC, and regression analysis). Furthermore, I recommend presenting the Bland-Altman results in a figure, not a table, since visual inspection, in this case, is more direct in interpreting results. Regression lines with the respective r2 and SEE values must also be shown to analyze the concurrent validity between devices.

**RESPONSE:**

Thank you for the suggestion to improve this section. We hope that our previous explanation regarding absolute measures of reliability has addressed this issue. Concerning the Bland-Altman results, we chose to present them in table format rather than as figures. This decision was made to streamline the manuscript and avoid excessive length (and the number of figures), while still ensuring that the results are clearly and comprehensively communicated. We hope that you understand the rationale behind our decision.

**COMMENT:**

DISCUSSION
- LL327: I recommend replacing the word "monitoring" with "measuring".

**RESPONSE:**

Thanks for the suggestion. The text has been revised accordingly.

**COMMENT:**
- LL330-331: "The results revealed that Vitruve and GymAware cannot be used interchangeably." If we consider the GymAware device as the gold standard, this sentence means that the Vitruve is not a valid device for measuring velocity and power. I suggest rewriting the sentence for clarity.

**RESPONSE:**

Thanks for the suggestion to clarify this point. We have rewritten the sentence to clearly state that Vitruve is not valid for measuring velocity and power:

“Considering GymAware as a reference point, Vitruve was not valid for measuring velocity and power outcomes. Acceptable validity was observed only for PV in the Smith machine back squat, while the rest of the variables were mostly inaccurate across relative loads and regardless of the exercise mode.”

**COMMENT:**
- LL342-344: According to these lines, only the measurement of PV during the Smith machine back squat seems valid. Therefore, the practical implications paragraph and the conclusions sentences should adequately address these results to clarify the readers. Vitruve is not valid for measuring MV during the back squat in both variants, but it is valid for measuring PV only during the Smith machine back squat.

**RESPONSE:**

Thanks for this appreciation. We have modified the text to specify in which context Vitruve met or not the validity criteria established:

“Considering GymAware as a reference point, Vitruve was not valid for measuring velocity and power outcomes. Acceptable validity was observed only for PV in the Smith machine back squat, while the rest of the variables were mostly inaccurate across relative loads and regardless of the exercise mode.”

**COMMENT:**
- LL347-348: "Several factors could be behind these underestimations of MV, and the higher validity observed for PV". "For MV, Vitruve did not meet the validity criteria for any of the relative loads during the free-weight back squat exercise mode or Smith machine back squat exercise mode». The sentence must be reformulated to clearly highlight this information.

**RESPONSE:**

Thank you for your comment. If the reviewer suggests that we should highlight that Vitruve underestimated the MV, it is later specified that a fixed negative bias was observed for all relative loads (range: -0.02 to -0.05 m/s) (Table 1).

**COMMENT:**
- LL349: It is said that the "GymAware ... includes an angle sensor that corrects the vertical displacement according to the horizontal motion of the lift". However, what is the influence of this sensor? Is it really that relevant?

**RESPONSE:**

Thank you for your comment. According to Pérez-Castilla et al., placing the LPT 15 cm away from the vertical projection of the barbell results in a reduction of 0.05 m/s in measurement accuracy. Therefore, it is important to control for this issue. This consideration might also explain the findings in studies such as Janicijevic et al., where GymAware was compared to the T-Force in the free-weight back squat. The study observed lower values with GymAware, which could be attributed to the correction for this horizontal component.

Pérez-Castilla, A., Miras-Moreno, S., García-Vega, A. J., & García-Ramos, A. (2022). The placement of linear transducers affects the magnitude but not the intra-session reliability of kinematic variables during the bench press exercise. Isokinetics and Exercise Science, 30(4), 357-366.

Pérez-Castilla, A., Miras-Moreno, S., García-Vega, A. J., & García-Ramos, A. (2022). The placement of linear transducers affects the magnitude but not the intra-session reliability of kinematic variables during the bench press exercise. Isokinetics and Exercise Science, 30(4), 357-366.

**COMMENT:**
- LL350: "Additionally, the data processing performed by the software of each device might cause a different start and end point selection of the concentric phase." How does the mechanism differ? In lines 213-214, the authors indicate, "Both linear position transducers internally collect and process the data in a similar fashion". Please reformulate for clarity.

**RESPONSE:**

Thank you for suggesting we clarify this point. When we mentioned that "Both linear position transducers internally collect and process the data in a similar fashion," we were referring to the general procedures of how an LPT operates, as indicated in the text below:

“Briefly, a retractable cable is fixed to the axis of an electromechanical sensor. The cable extension produces the rotation of the transducer axis, which converts it into pulses and then to linear displacement. Thereafter, the double differentiation of displacement data permits the calculation of acceleration, from which all the variables reported by these devices are calculated (11)”

However, these devices may yield different measurements, such as mean velocity or power, due to variations in how they determine the start and end of the movement. The software in these devices may use different threshold criteria to distinguish between minor vibrations or noise and actual movement, which can lead to discrepancies in the data collected. The text has been reformulated for clarity:

“Additionally, although both devices work through the same procedures (i.e., the cable extension produces the rotation of the transducer axis, which converts it into pulses and then to linear displacement), each software may use different threshold criteria to distinguish between minor vibrations or noise and actual movement, which can lead to discrepancies in the data collected.”

**COMMENT:**
- I recommend that the authors address some of the study's limitations. For example, some limitations include the low sample size, the absence of velocity data for some relative loads (30, 50, and 70% 1RM), and the lack of a gold standard device such as a 3D motion capture system to measure velocity. Furthermore, if power variables are maintained in the analysis, the lack of a direct force measurement to determine power must be recognized.

**RESPONSE:**

Thanks for the suggestion and we completely agree. A limitation paragraph has been added to the text:

A few limitations should be acknowledged. The absence of data for certain relative loads (30%, 50%, and 70% 1RM) may impact the comprehensiveness of our analysis. Although the validity of GymAware has been well established in several studies (12–14), Vitruve has not been compared to a gold-standard device, such as a 3D motion capture system. The same considerations apply to the power variables included, as their validity with a gold-standard device (i.e., force platform) has not been analyzed either.

Since the post-hoc power analysis revealed a statistical power of 0.93, the sample size was not considered as a limitation.

**COMMENT:**
- I also recommend that the authors provide future research lines on this topic and practical recommendations for coaches and researchers regarding using the Vitruve device in the free-weight and Smith machine back squat. What should and should not be used must be mentioned.

**RESPONSE:**

Thanks for the comment. Future research lines have been suggested at the end of the limitation paragraph:

“Future research lines should address these knowledge gaps, as well as the analysis of the validity and reliability of Vitruve in other exercises (e.g., bench press, deadlift).”

Solving some of the previous comments, the conclusion section has been reinforced with practical recommendations for coaches and researchers regarding what should and should not be done when using Vitruve:

“Considering GymAware as a reference point, Vitruve was not valid for measuring velocity and power outcomes. Acceptable validity was observed only for PV in the Smith machine back squat, while the rest of the variables were mostly inaccurate across relative loads and regardless of the exercise mode. On the other hand, Vitruve users should note that MV and PV displayed superior reliability compared to MP and PP. Additionally, all variables measured by Vitruve were more reliable in the Smith machine compared to the free-weight back squat exercise mode. Finally, practitioners are encouraged to consider the SDD reported here for MV, PV, MP, and PP when interpreting meaningful changes in performance.”
