# Supplementary material for: Validity and reliability of velocity and power measures provided by the Vitruve linear position transducer
Source: PLoS One. 2024 Oct 24;19(10):e0312348. doi: 10.1371/journal.pone.0312348 (PMC11500865; doi:10.1371/journal.pone.0312348)
Supplement: Attachment — Submitted filename: Response to reviewers.docx [file pone.0312348.s004.docx]

**Validity and Reliability of Velocity and Power Measures Provided by the Vitruve Linear Position Transducer**

**Reviewer #1:**

**COMMENT:**

Thank you, some of the claims are better. However, when using words like "superior" it makes it sound like something is actually good. If you eat pizza and the taste of one pizza is 4/10 and the other is 5/10, the second is superior than the other. Despite being superior, would you want to eat that pizza? No. I feel the same with this paper... we have tons of devices to choose from, some of which are excellent at different loads, different exercises, with machines, with free weights etc. Yet, here we have a device that isn't as good (according to the data presented). So yea, although the data show the Vitruve is better at measuring V than P, that it's better in a machine than with a free weight, it seems to be a poor choice for people who will actually use this. Remember, even if you hedge your statements a bit, authors will read your title, abstract, and conclusion and still think this device is good (it's just not excellent at everything). However, compared to the other options out there, this device doesn't seem to be up to par yet. In no way am I involved with any company, I don't want to promote anything, and I don't want to convince you (or potential readers) what devices they should or shouldn't use. However, when looking at this data, and when looking at other similar data on other devices, these data indicate that the device shouldn't be used in its current state. If you agree, please make some more changes throughout the document, especially at strategic points. However, if you have a different opinion and you feel as though you want to highlight the positive aspects of the data (compared to the overall negative aspects), I cannot stop you because it's just the way you choose to report and interpret the data. There would not be anything scientifically wrong, per se.

**RESPONSE:**

First of all, the authors would like to thank the reviewer for their constructive comments. The comments have significantly improved the quality of the manuscript and we are grateful for that.

Regarding the suggestion to adopt a more conservative tone in certain sections of the manuscript, we would like to address this concern. The term "superior" is specifically used when comparing exercise modes (Smith vs. Free), and we believe that readers will understand this distinction is not a claim of good performance relative to our reference system. Nevertheless, we have taken the reviewer's perspective into account and rewritten the conclusion as follows:

*Considering GymAware as a reference point, the Vitruve system demonstrated insufficient validity for measuring velocity and power outcomes. Acceptable validity was observed only for PV in the Smith machine back squat, while relative loads and other exercise modes yielded inaccurate results. Conversely, users of the Vitruve system should note that MV and PV exhibited greater reliability compared to MP and PP. Additionally, all variables measured by Vitruve showed higher reliability in the Smith machine than in the free-weight back squat exercise mode. If practitioners choose to use the Vitruve device despite its poor validity relative to GymAware, they are encouraged to pay special attention to the SDD reported in this study for the velocity and power metrics obtained in both the free-weight and Smith machine back squat exercises. This consideration is essential for accurately interpreting meaningful changes in performance.*

In this manner, we aim to clarify that we, the authors, are not recommending the use of Vitruve; rather, we present statements based on the results of its validity and reliability analysis. We hope we have made this clearer now, and again, we really appreciate your comments on this.

We also amended the “conclusion” section of the abstract as follows: *Acceptable validity was observed only for PV in the Smith machine back squat, while the other variables—regardless of relative loads and exercise modes—were mostly inaccurate. All variables demonstrated acceptable reliability, with greater reliability noted in the Smith machine compared to the free-weight back squat exercise mode.*

We really hope that the changes made address your comments.
